# Supplementary material for: Homologous recombination changes the context of Cytochrome b transcription in the mitochondrial genome of Silene vulgaris KRA
Source: BMC Genomics. 2018 Dec 4;19:874. doi: 10.1186/s12864-018-5254-0 (PMC6280394; doi:10.1186/s12864-018-5254-0)
Supplement: Supplementary file 1 — Figure S1. Maps of S. vulgaris KRA mitochondrial chromosomes. (PDF 5577 kb) [file 12864_2018_5254_MOESM1_ESM.pdf]

Figure S1. Maps of *S. vulgaris* KRA mitochondrial chromosomes. Boxes inside and outside the circle correspond to genes on the clockwise and anti-clockwise strand, respectively. GC content is indicated by the inner ring (shown only for chromosomes 1 -3).

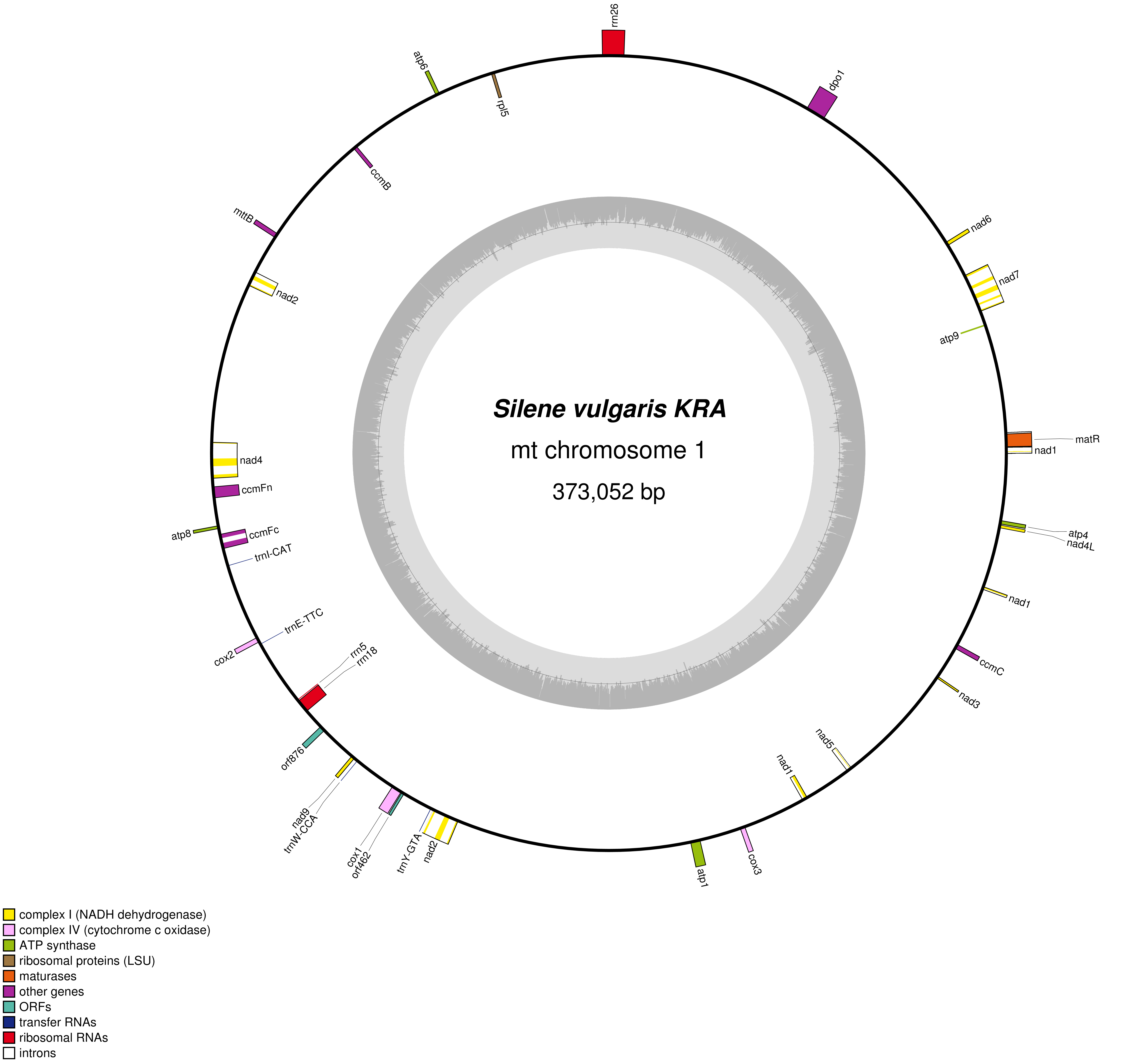

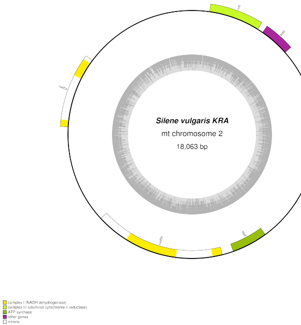

Silene vulgaris KRA
mt chromosome 2
18,063 bp
complex I (NADH dehydrogenase)
complex III (ubichinol cytochrome c reductase)
ATP synthase
other genes
introns

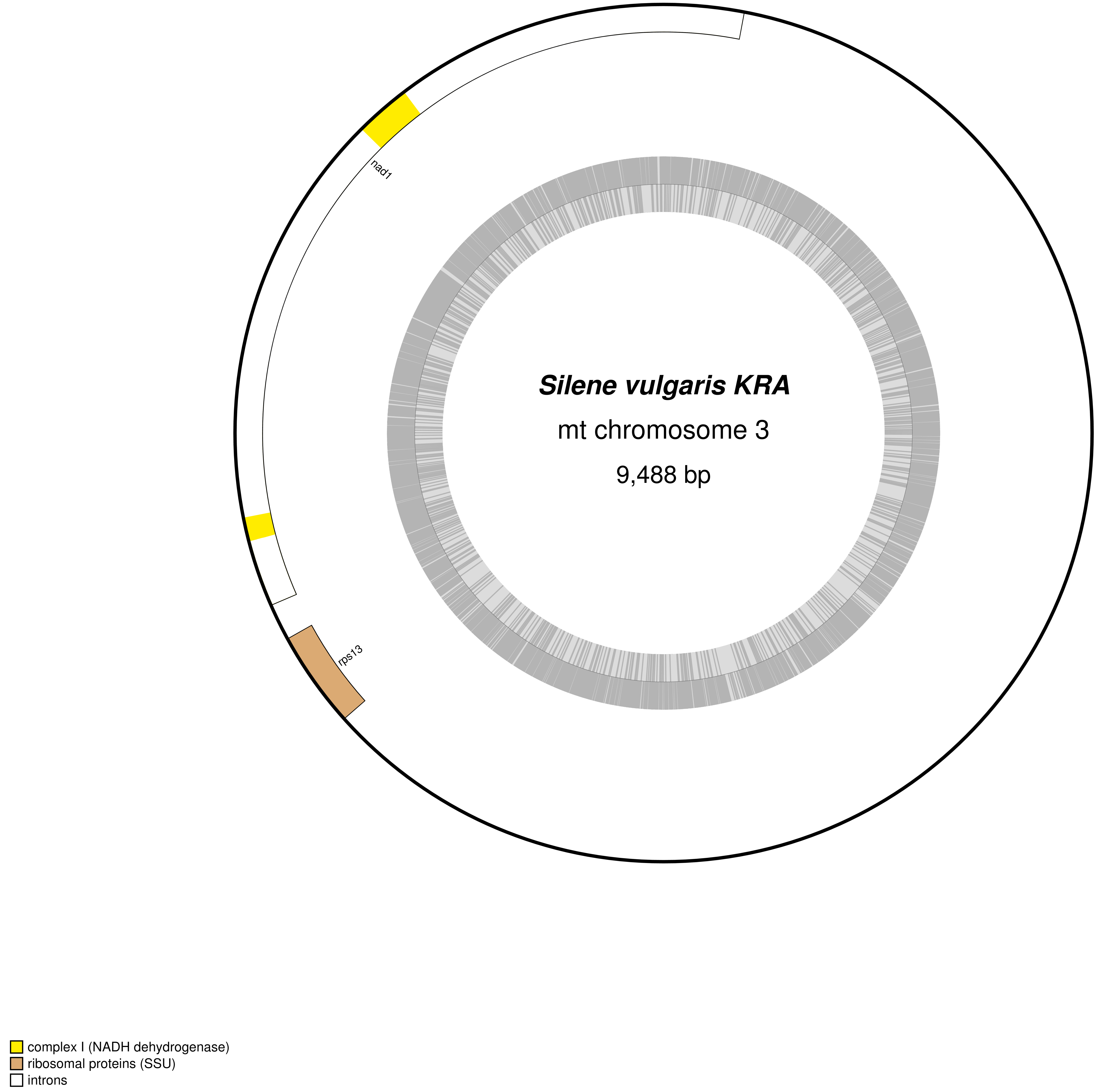

Silene vulgaris KRA
mt chromosome 3
9,488 bp
nad1
rps13
complex I (NADH dehydrogenase)
ribosomal proteins (SSU)
introns

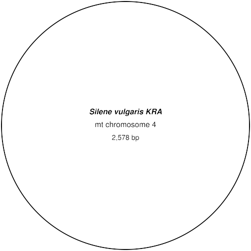
Silene vulgaris KRA
mt chromosome 4
2,578 bp

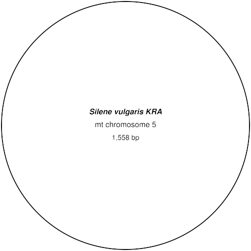
Silene vulgaris KRA
mt chromosome 5
1,558 bp
