# Supplementary material for: Homologous recombination changes the context of Cytochrome b transcription in the mitochondrial genome of Silene vulgaris KRA
Source: BMC Genomics. 2018 Dec 4;19:874. doi: 10.1186/s12864-018-5254-0 (PMC6280394; doi:10.1186/s12864-018-5254-0)
Supplement: Supplementary file 2 — Figure S2. Phylogenetic relationships among five mitochondrial genomes of S. vulgaris. (PDF 50 kb) [file 12864_2018_5254_MOESM2_ESM.pdf]

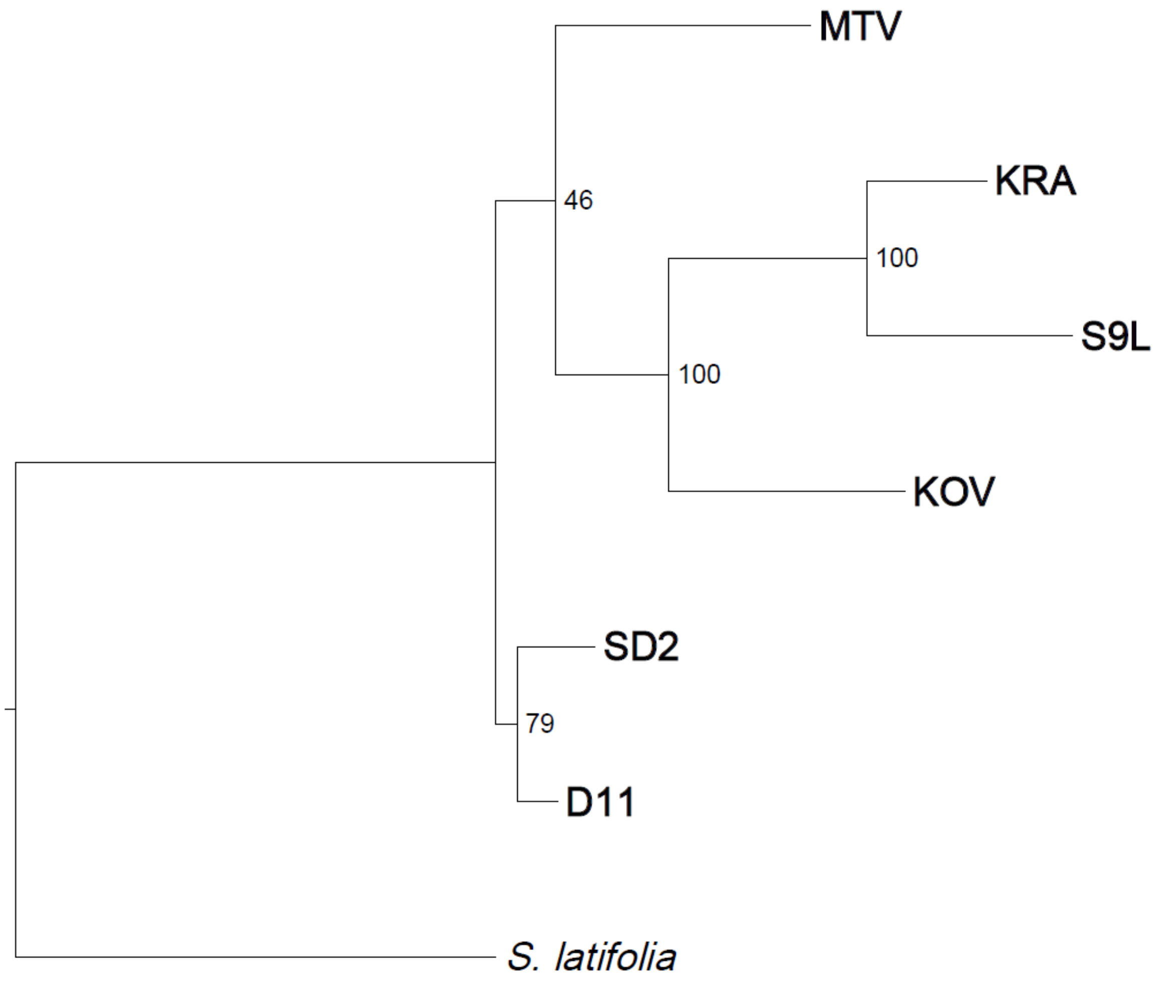

Figure S2. Phylogenetic tree of *S. vulgaris* mt haplotypes. *S. vulgaris* KRA mt genome is closely related to the S9L mt genome. A bootstrapped maximum likelihood phylogenetic tree was generated with RAxML using concatenated protein sequences from completely sequenced *S. vulgaris* mt genomes.
