# Supplementary material for: Homologous recombination changes the context of Cytochrome b transcription in the mitochondrial genome of Silene vulgaris KRA
Source: BMC Genomics. 2018 Dec 4;19:874. doi: 10.1186/s12864-018-5254-0 (PMC6280394; doi:10.1186/s12864-018-5254-0)
Supplement: Supplementary file 3 — Table S1. Nucleotide polymorphism in the atp6 gene. (DOCX 13 kb) [file 12864_2018_5254_MOESM3_ESM.docx]

**Table S1.** Nucleotide polymorphism in *atp6*. Twenty-one sites variable among five *S. vulgaris* mt genomes are shown. Dots indicate sequence identity with the *S. vulgaris* KOV/MTV/SD2 haplotype. The positions of unique substitutions in *S. vulgaris* KRA are in red color.

|  | 354 | 392 | 399 | 402 | 403 | 405 | 406 | 409 | 410 | 414 | 418 | 426 | 496 | 504 | 525 | 602 | 603 | 604 | 915 | 916 | 1068 |
| --- | --- | --- | --- | --- | --- | --- | --- | --- | --- | --- | --- | --- | --- | --- | --- | --- | --- | --- | --- | --- | --- |
| *S. vulgaris* KOV/MTV/SD2 | T | C | C | A | T | G | A | C | C | G | C | C | C | G | C | G | G | C | G | T | A |
| *S. vulgaris* KRA | **C** | **.** | **.** | **.** | **.** | **.** | **.** | **.** | **.** | **.** | **.** | **G** | **.** | **.** | **.** | **.** | **.** | **.** | **C** | **C** | **T** |
| *S. vulgaris* S9L | . | A | T | T | C | T | T | G | A | C | T | **.** | T | T | G | T | T | A | . | . | . |
| S. *latifolia* | **.** | **.** | **.** | **.** | **.** | **.** | **.** | **.** | **.** | **.** | **.** | **.** | **.** | **.** | **.** | **.** | **.** | **.** | **.** | **.** | **.** |
| *S. noctiflora* | G | . | T | T | C | T | T | G | A | C | **.** | **.** | **.** | T | **.** | **.** | C | **.** | T | . | **.** |
| *S. conica* | **.** | **.** | **.** | T | **.** | **.** | **.** | **.** | **.** | **.** | **.** | **.** | **.** | **.** | A | **.** | A | **.** | T | . | **.** |
| *Beta vulgaris* | C | **.** | **.** | **.** | **.** | **.** | **.** | **.** | **.** | **.** | A | **.** | **.** | T | **.** | **.** | T | **.** | T | **.** | **.** |
| *Vitis vinifera* | C | **.** | **.** | **.** | **.** | **.** | **.** | **.** | **.** | **.** | A | **.** | **.** | T | **.** | **.** | T | **.** | T | **.** | **.** |
