# Supplementary material for: Homologous recombination changes the context of Cytochrome b transcription in the mitochondrial genome of Silene vulgaris KRA
Source: BMC Genomics. 2018 Dec 4;19:874. doi: 10.1186/s12864-018-5254-0 (PMC6280394; doi:10.1186/s12864-018-5254-0)
Supplement: Supplementary file 4 — Figure S3. The alignment of the small mitochondrial autonomous chromosomes from five genomes of S. vulgaris. (PDF 61 kb) [file 12864_2018_5254_MOESM4_ESM.pdf]

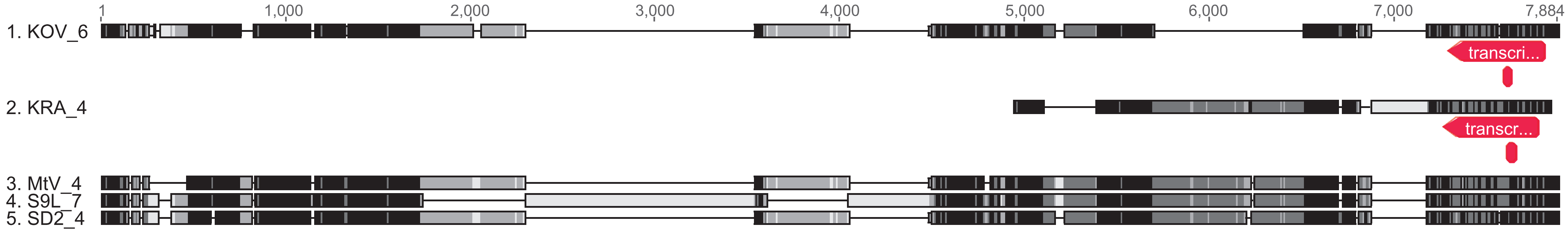

Figure S3. The alignment of small autonomous chromosome from five completely sequenced mt genomes of *S. vulgaris*. The occasionally transcribed region and putative hairpin structure in the KRA and KOV mt genomes are indicated by red color.
