# Supplementary figures and images for: Homologous recombination changes the context of Cytochrome b transcription in the mitochondrial genome of Silene vulgaris KRA
Source: BMC Genomics. 2018 Dec 4;19:874. doi: 10.1186/s12864-018-5254-0 (PMC6280394; doi:10.1186/s12864-018-5254-0)

**Figure S4.** The coverages of five genes in *S. vulgaris* KRA: *rpl5* (a), *nad5* (b), *matR* (c), *cob* (d), *bobt* (e).

a

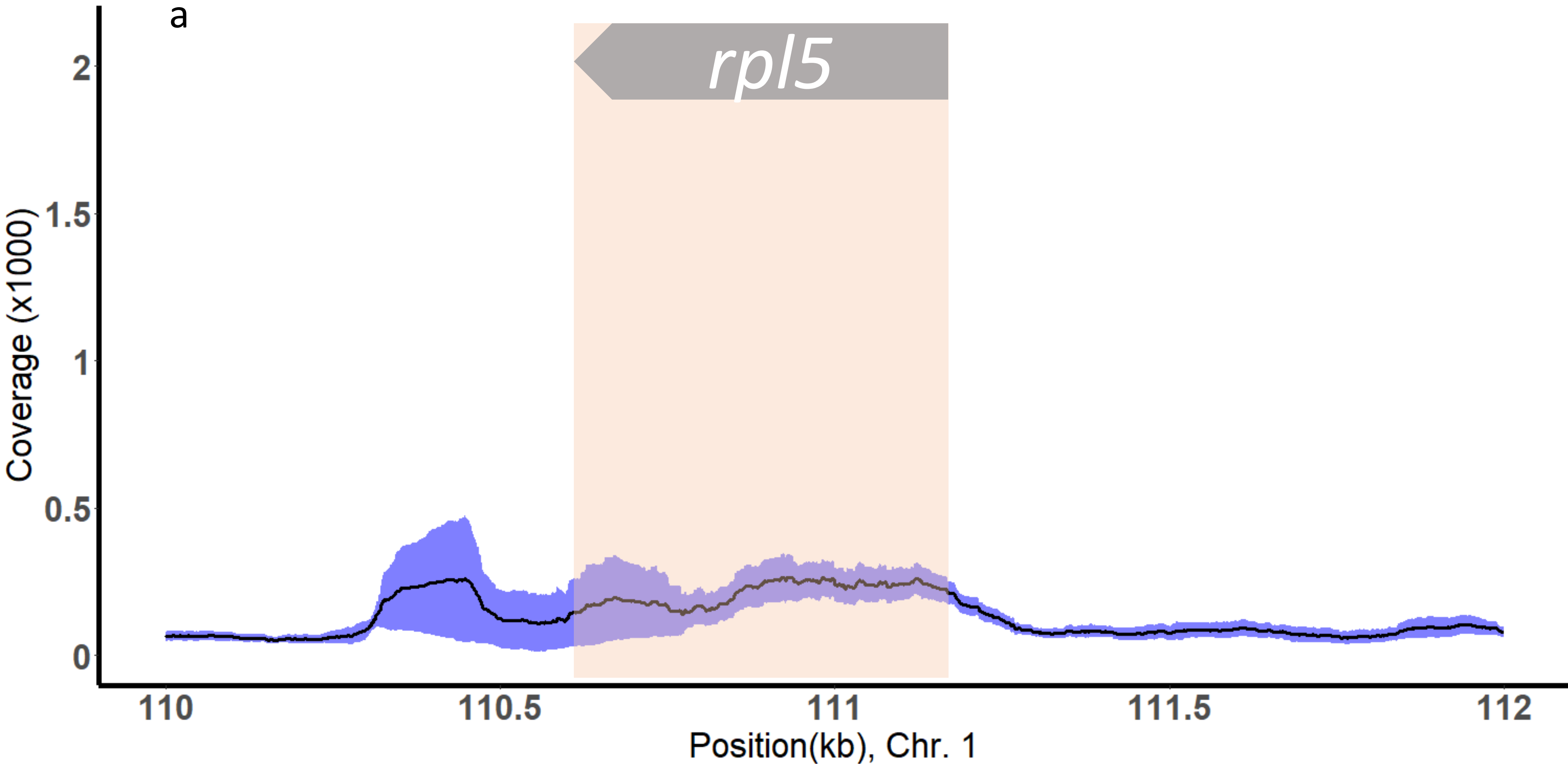

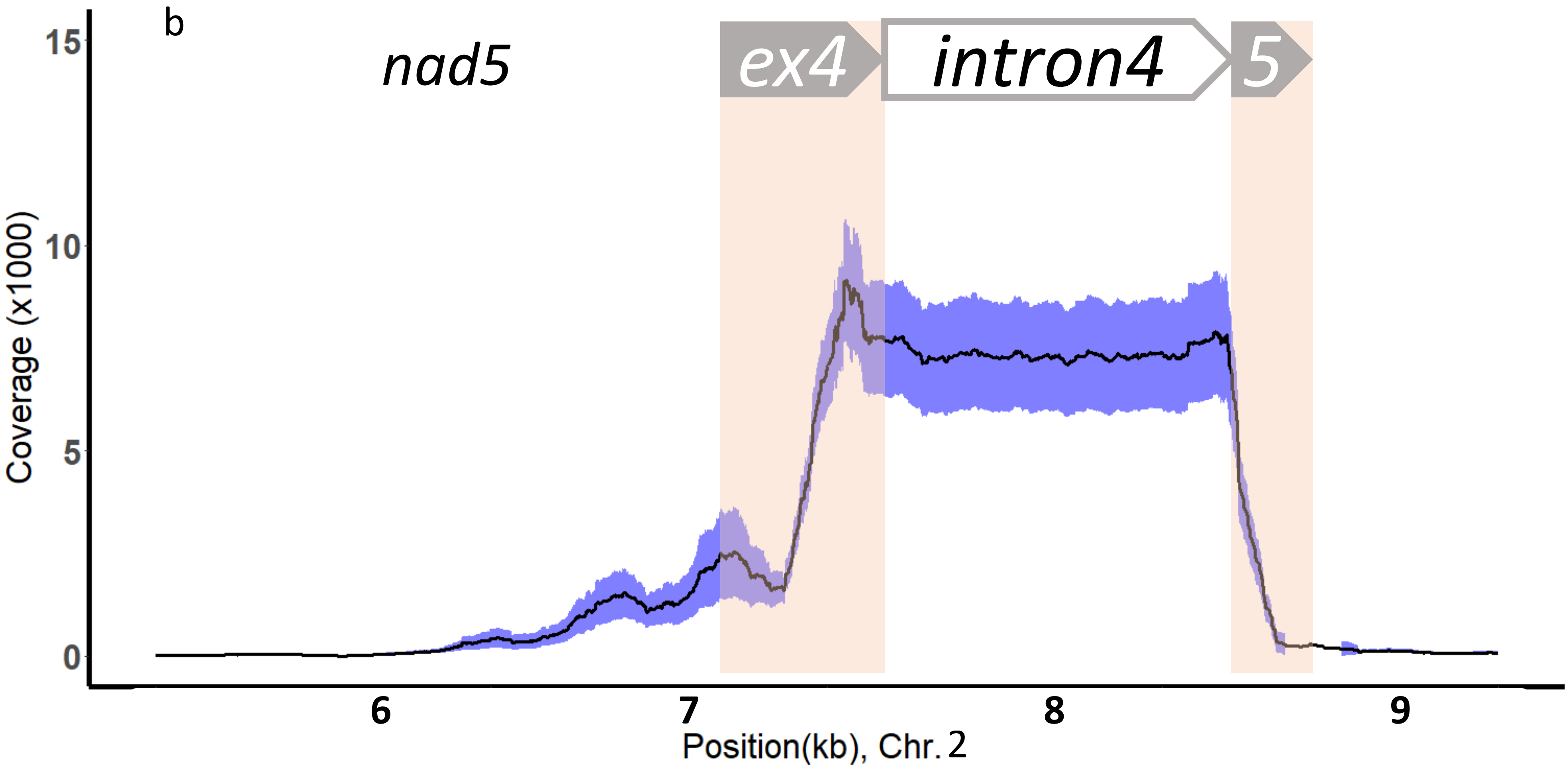

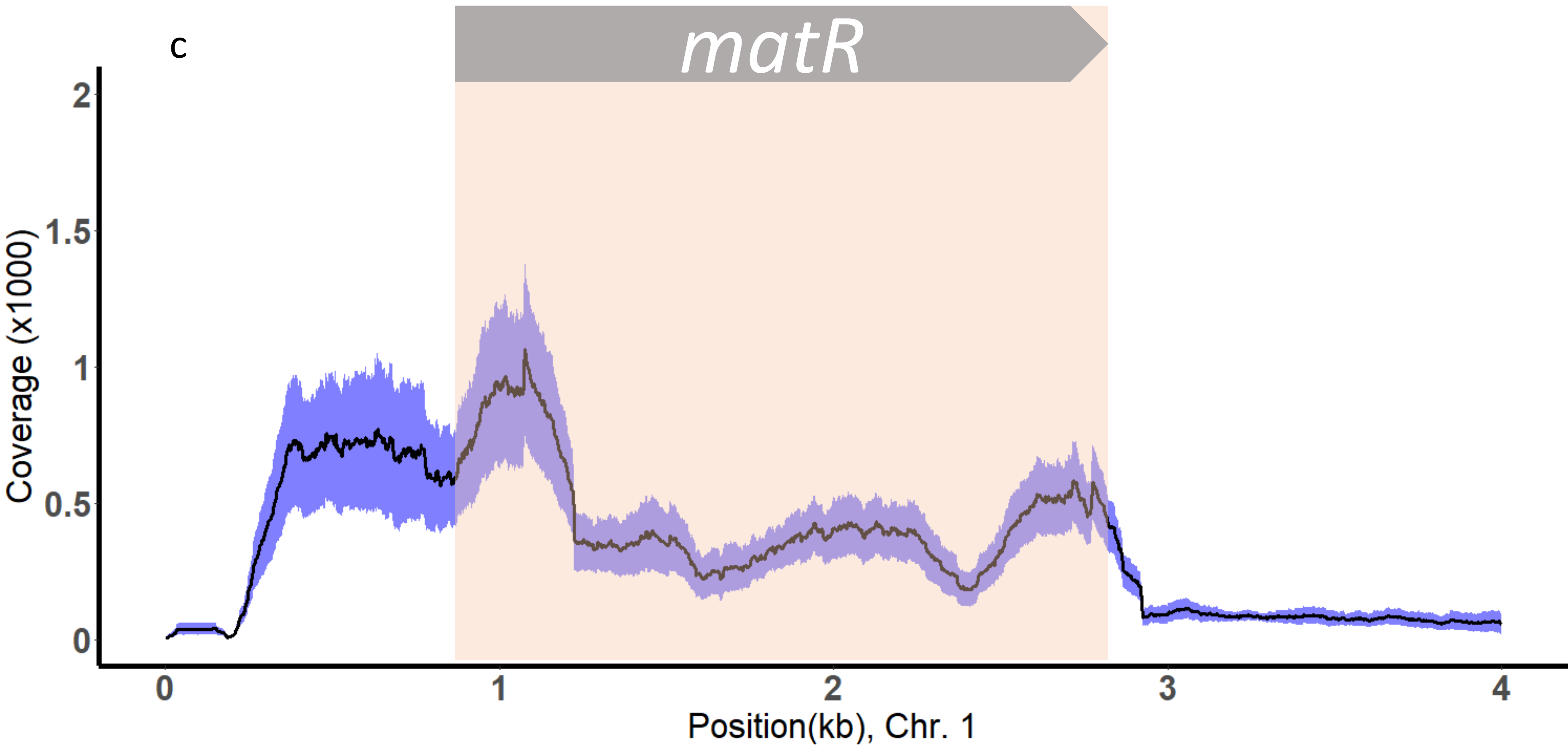

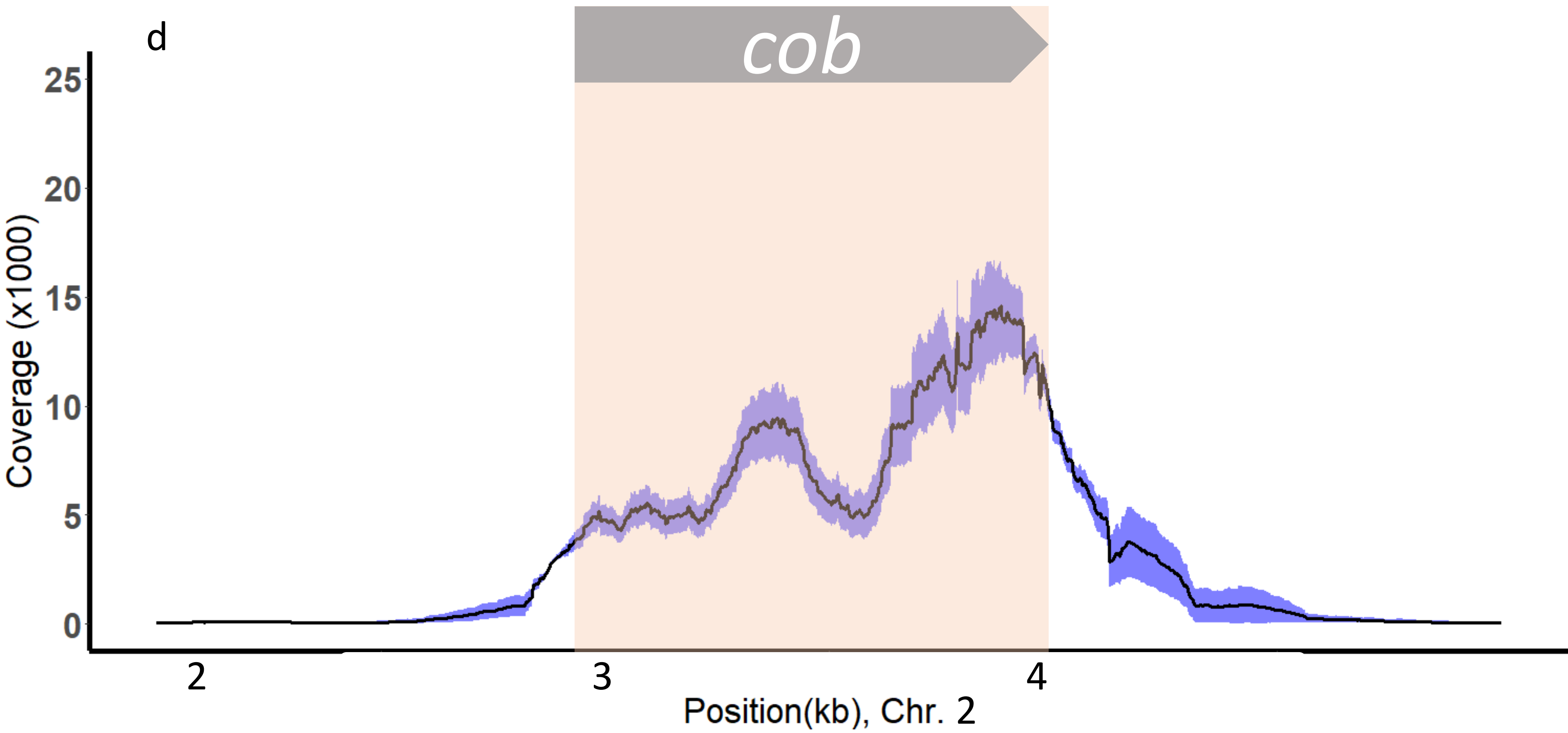

e

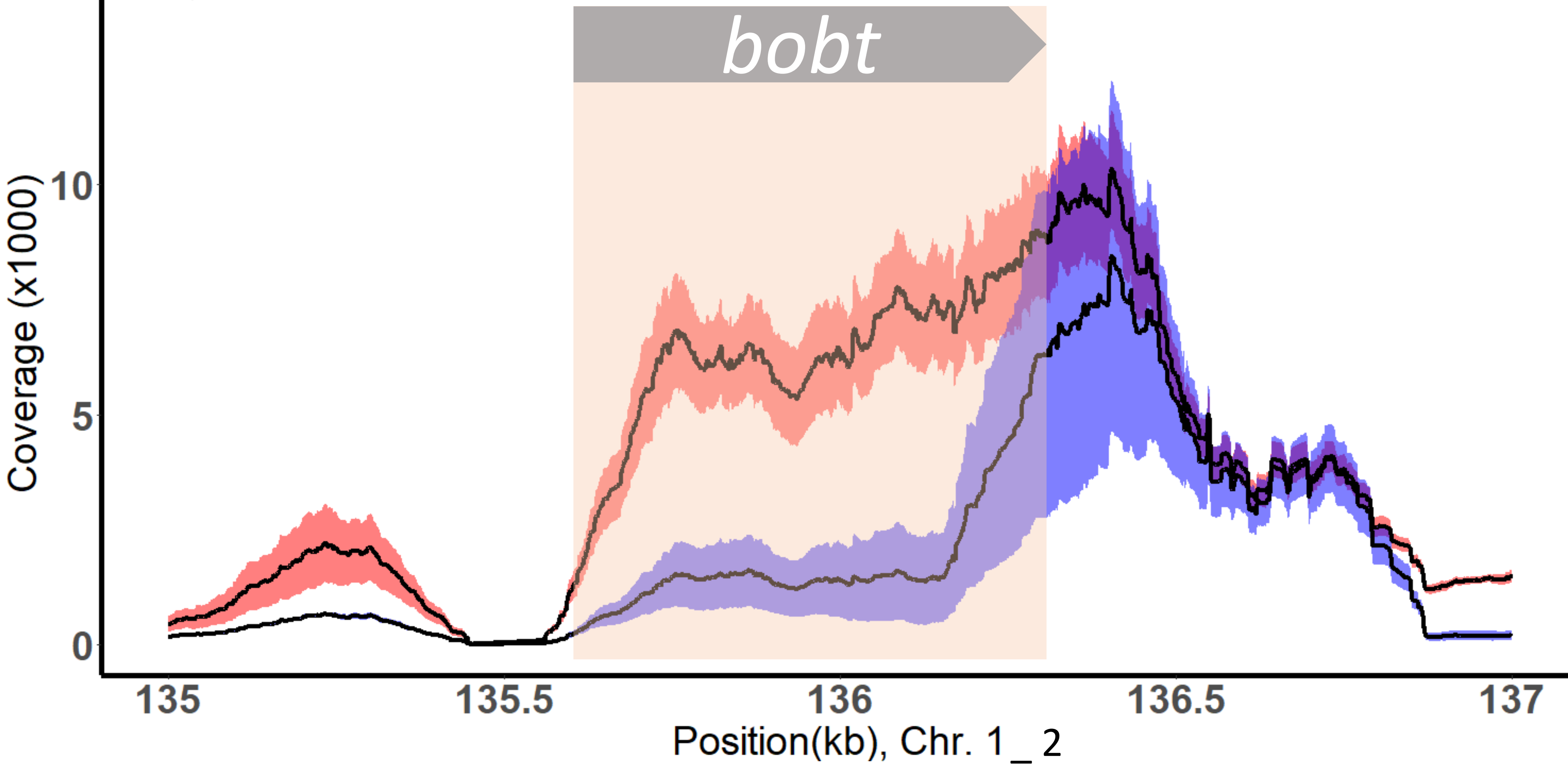

Supplement: Supplementary file 5 — Figure S4. Coverage of selected mitochondrial genes of S. vulgaris KRA. (PDF 164 kb) [file 12864_2018_5254_MOESM5_ESM.pdf]
