# Supplementary material for: Homologous recombination changes the context of Cytochrome b transcription in the mitochondrial genome of Silene vulgaris KRA
Source: BMC Genomics. 2018 Dec 4;19:874. doi: 10.1186/s12864-018-5254-0 (PMC6280394; doi:10.1186/s12864-018-5254-0)
Supplement: Supplementary file 6 — Figure S5. Recombination across the cob repeat visualized by Southern hybridization with EcoRI digested DNA. (PDF 37 kb) [file 12864_2018_5254_MOESM6_ESM.pdf]

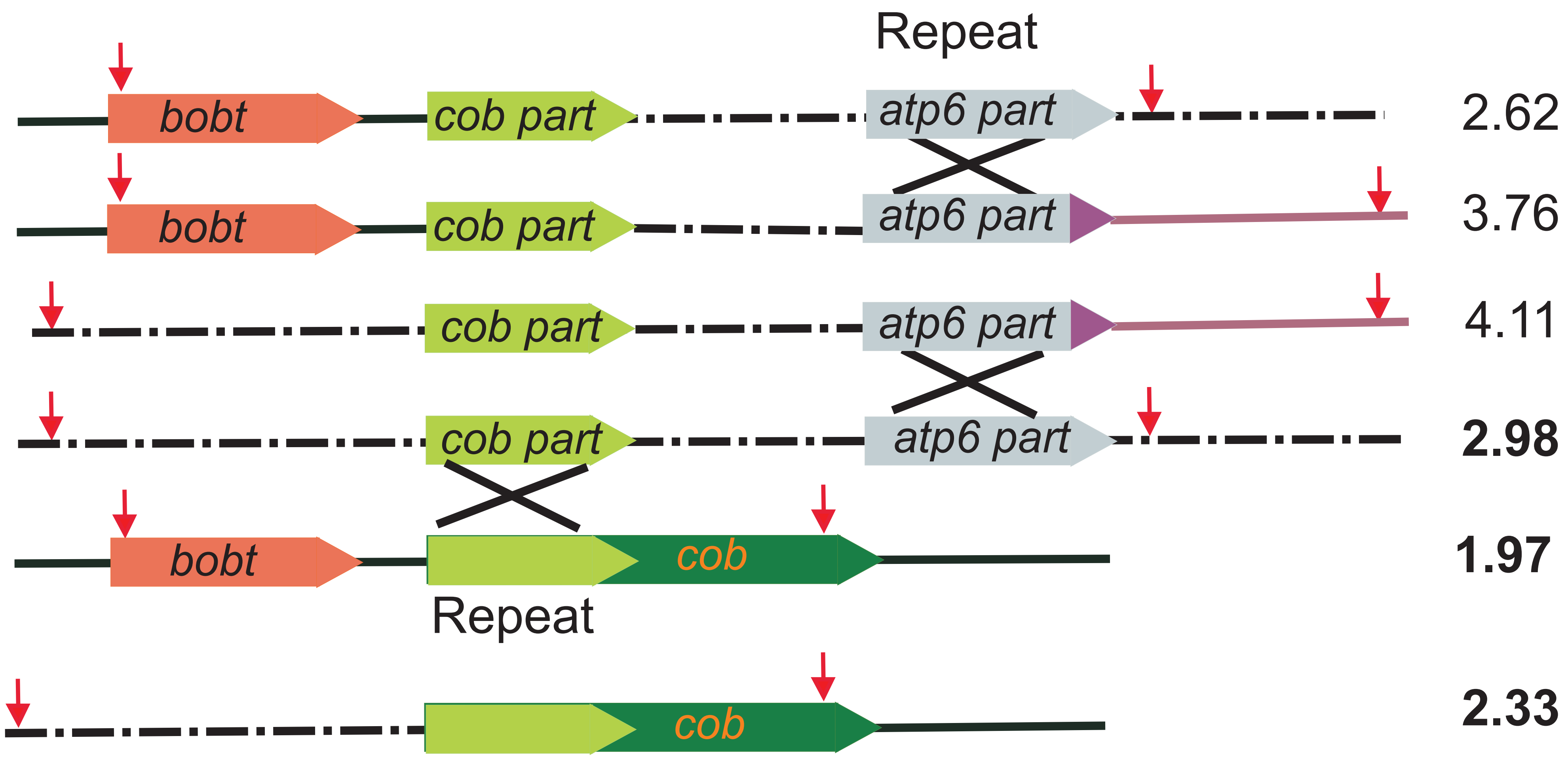

Figure S5. Recombination across *cob* repeat. The positions of the *Eco*RI sites in the vicinity of *cob* sequences are shown for six recombinant configurations. Southern hybridization with the *cob* probe is shown on the right. Total DNA was extracted from leaves of female and hermaphroditic plants and digested with *Eco*RI. The fragment sizes corresponding to the respective recombinant configurations are given in kb. The molecular standard in kb is shown on the right.
