# Supplementary material for: Homologous recombination changes the context of Cytochrome b transcription in the mitochondrial genome of Silene vulgaris KRA
Source: BMC Genomics. 2018 Dec 4;19:874. doi: 10.1186/s12864-018-5254-0 (PMC6280394; doi:10.1186/s12864-018-5254-0)
Supplement: Supplementary file 7 — Figure S6. Summary of chimeric ORFs > 300 bp in length in the mitochondrial genome of S. vulgaris KRA. (PDF 24 kb) [file 12864_2018_5254_MOESM7_ESM.pdf]

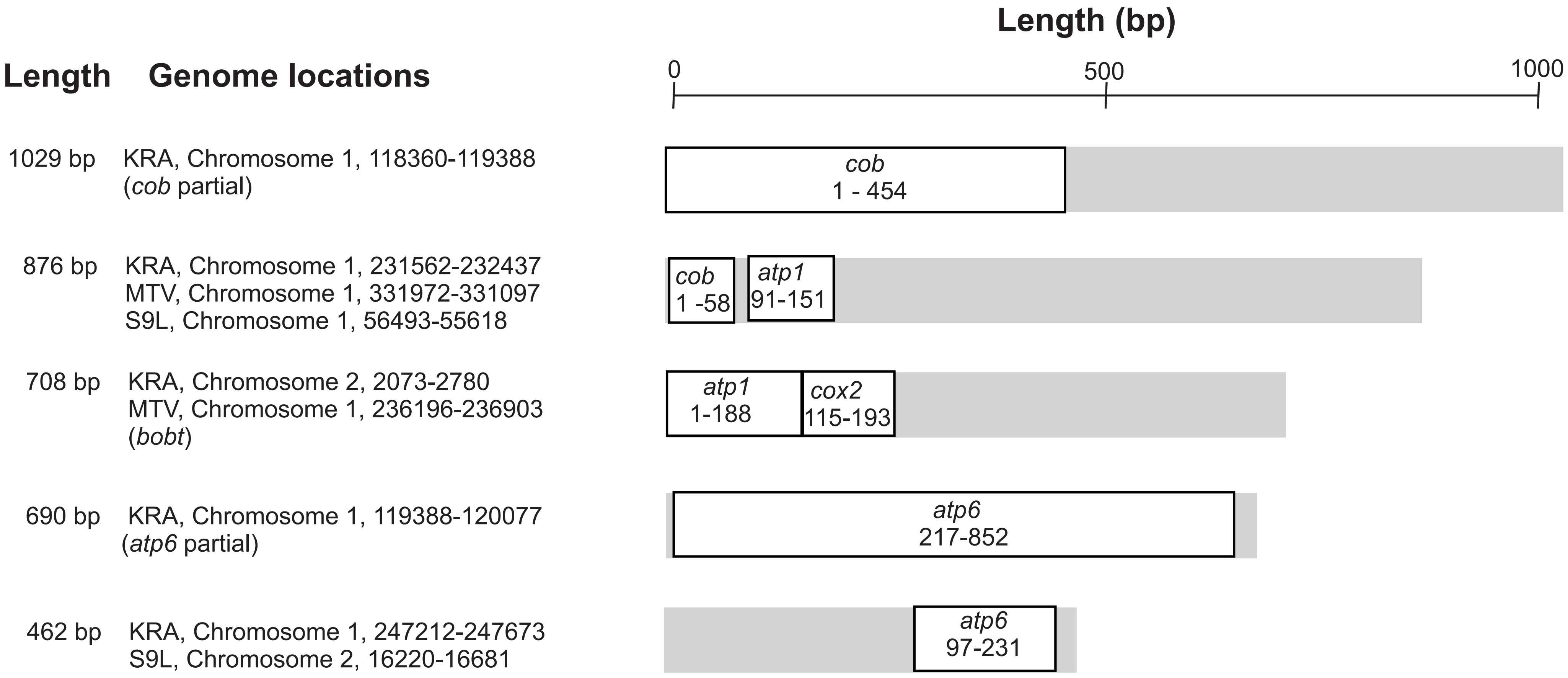

Figure S6. Summary of chimeric ORFs > 300 bp in length in the mt genome of *S. vulgaris* KRA. White boxes show the fragments of mitochondrial protein genes with corresponding nucleotide positions noted in the box. Grey regions indicate sequence of unknown origin. The presence of the same chimeric ORFs in additional mt genomes of *S. vulgaris* is reported. The synonymous name is written in parentheses.
