# Supplementary material for: Homologous recombination changes the context of Cytochrome b transcription in the mitochondrial genome of Silene vulgaris KRA
Source: BMC Genomics. 2018 Dec 4;19:874. doi: 10.1186/s12864-018-5254-0 (PMC6280394; doi:10.1186/s12864-018-5254-0)
Supplement: Supplementary file 9 — Figure S7. Comparison of editing extent between S. vulgaris KRA and KOV. (PDF 215 kb) [file 12864_2018_5254_MOESM9_ESM.pdf]

a

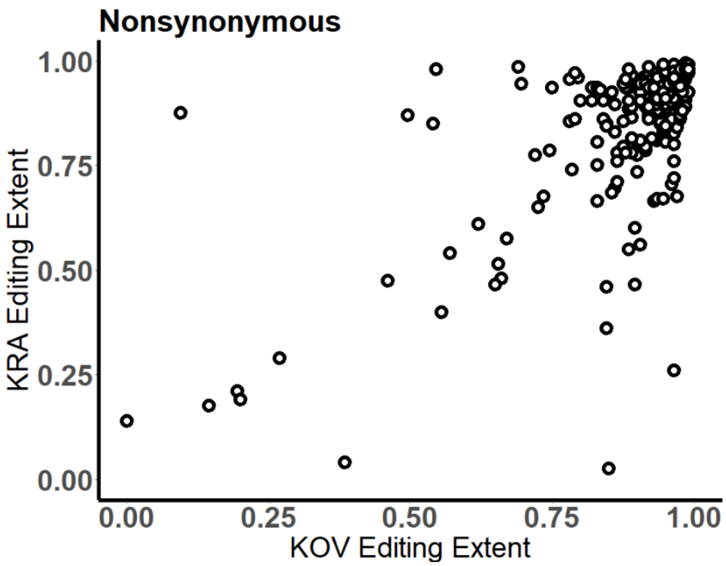

b

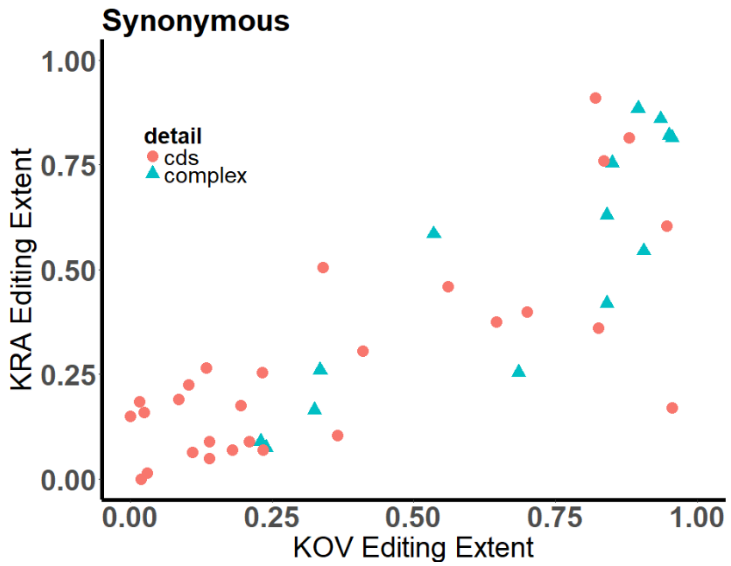

c

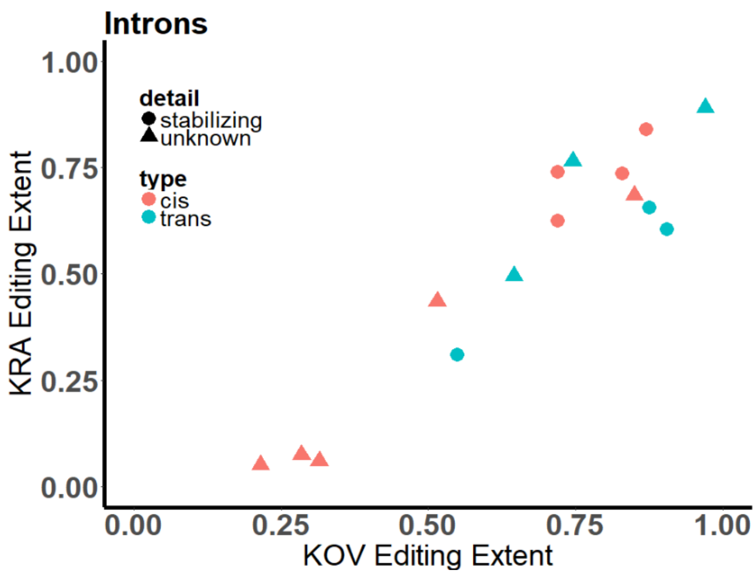

Figure S7. The comparison of editing extent between *S. vulgaris* KRA and KOV. Editing in non-synonymous sites of protein/coding genes (a), editing in synonymous sites of protein/coding genes (b), editing in itrons (c). Abbreviations: cds – amino acid is always changed by editing; complex – editing changes coding capacity depending on the adjacent nucleotide; cis – *cis* spliced intro; trans – *trans* spliced intron
